# Cumulative intraoperative oxygen exposure acts as a predictor for postoperative acute exacerbation of interstitial lung disease: a retrospective cohort study

**DOI:** 10.1186/s12890-026-04482-x

**Published:** 2026-07-17

**Authors:** Ryotaro Katsuya, Naoki Ozeki, Tomohito Okubo, Kiichi Tatakawa, Chihiro Furuta, Hideaki Note, Toyofumi Fengshi Chen-Yoshikawa, Takayuki Fukui

**Affiliations:** 1 Division of Chest Surgery, Department of Surgery, Aichi Medical University, 1-1 Yazakokarimata, Nagakute, Aichi 480-1195 Japan; 2 Department of Thoracic Surgery, Nagoya University Graduate School of Medicine, Nagoya, Japan; 3 Department of Anesthesia, Aichi Medical University, Nagakute, Japan

**Keywords:** Oxygen, Pulmonary function, Acute exacerbation, Interstitial lung disease

## Abstract

**Background:**

Excessive oxygen administration during surgery may induce lung damage via oxidative stress; however, limited evidence exists on how intraoperative oxygen usage and preoperative pulmonary function predict acute exacerbation (AE) in patients with interstitial lung disease (ILD). This study evaluated whether intraoperative oxygen use and preoperative pulmonary function could predict postoperative AEs in patients with ILD who underwent lung surgery.

**Methods:**

We retrospectively analyzed 78 patients with ILD who underwent lung surgery between 2014 and 2022. Clinical variables, including intraoperative oxygen usage and preoperative pulmonary function tests, were assessed for their association with AEs occurring within 1 month (1mAE) and 1 year (1yAE) postoperatively using Firth’s bias-reduced logistic regression.

**Results:**

Eight patients (10%) had 1mAE, and 14 (18%) had 1yAE. Multivariable analysis showed that lower %vital capacity (%VC) consistently predicted both 1mAE and 1yAE, whereas lower %diffusing capacity for carbon monoxide (%DLCO) significantly predicted only 1yAE. Notably, total oxygen administration > 990 L exhibited a marginal association with 1mAE (OR 2.12, 95% CI: 0.93–4.99, *p* = 0.071). Even after adjusting for operative and anesthesia durations, total oxygen volume remained a significant independent predictor for 1yAE (OR 2.29, 95% CI: 1.09–4.93, *p* = 0.029). No significant associations were found between AEs and factors such as the mean fraction of inspired oxygen (FiO_2_), forced expiratory volume in 1 s, sex, preoperative steroid use, computed tomography patterns, serum Krebs von den Lungen-6 levels, or surgical procedures.

**Conclusions:**

Cumulative intraoperative oxygen administration (rather than mean FiO2) serves as an independent predictor of long-term postoperative AEs in patients with ILD. These findings suggest that cumulative oxygen exposure may contribute to long-term postoperative risk through mechanisms related to oxidative stress.

**Trial registration:**

Aichi Medical University 2025-011.

## Background

Acute exacerbation (AE) of interstitial lung disease (ILD) is a serious postoperative complication of pulmonary surgery and is associated with high morbidity and mortality rates [1]. This condition is distinctively characterized by an abrupt and profound deterioration of respiratory function, often developing rapidly and unexpectedly in the postoperative period, and is consistently associated with dramatically elevated rates of both short- and long-term morbidity and mortality [2]. Previous studies have mainly focused on preoperative pulmonary function, computed tomography (CT) imaging patterns, and perioperative management strategies as risk factors for AE [1, 3–6].

AE of ILD is a serious surgical complication that is not limited to thoracic surgery. The associated mortality rate is extremely high [7–9]. Among patients with ILD, those undergoing lung resection with one-lung ventilation are considered to be at particularly high risk of AE [6], which further complicates the clinical management of such cases. Even when lung resection is unavoidable in patients with ILD, stratifying the postoperative risk of AE may allow for more vigilant management and earlier AE detection.

Various studies have examined postoperative risk stratification using risk scores, positron emission tomography imaging [4], and serum biomarkers [2, 10, 11]; however, few have focused on intraoperative factors. To the best of our knowledge, no previous study has specifically investigated the volume of oxygen administered during surgery as a risk factor. A decrease in % diffusing capacity for carbon monoxide (%DLCO) indicates a reduction in the alveolar-capillary surface area, helps evaluate the severity of ILD, and correlates with the malignancy grade of the developing lung adenocarcinoma [12].

Therefore, we retrospectively reviewed the anesthetic records of patients with ILD who underwent lung resection to investigate the potential impact of intraoperative factors on the development of AE. Additionally, we re-evaluated previously reported risk factors to assess their predictive value in our cohort.

Moreover, evidence regarding intraoperative oxygen administration protocols is limited, and the impact of preoperative pulmonary function on postoperative AE risk remains unclear. Oxidative stress induced by high-oxygen exposure during surgery has been suggested to contribute to lung injury [13]. Additionally, preoperative pulmonary function, specifically %vital capacity (%VC), %forced expiratory volume in 1 s (%FEV1), and %DLCO, may provide insights into the lungs’ ability to tolerate surgical stress. Understanding these associations is critical for optimizing perioperative management and minimizing postoperative complications in patients with ILD.

In this study, we conducted a retrospective analysis to evaluate whether intraoperative oxygen use and preoperative pulmonary function could predict postoperative AEs in patients with ILD who underwent lung surgery. By analyzing the impact of anesthesia-related factors and pulmonary function on AE during surgery, we anticipate improvements in perioperative management optimization and risk stratification.

## Materials and methods

### Study design and population

We conducted a retrospective study of patients with ILD who underwent pulmonary surgery at our institution between January 2014 and December 2022. Data were extracted from electronic medical records and included demographic, clinical, surgical, and follow-up information. This study was performed in accordance with the Declaration of Helsinki. The study protocol was approved by the Ethics Committee of Aichi Medical University (Approval No. 2025-011). The requirement for informed consent was waived by the Ethics Committee of Aichi Medical University due to the retrospective nature of the study. All data were anonymized to ensure patient confidentiality.

### Data collection

Patient information included age, sex, body mass index, and smoking history (current, former, or never smoker). Preoperative comorbidities, such as asthma and chronic obstructive pulmonary disease (COPD) were recorded. Preoperative evaluations included pulmonary function tests, serum Krebs von den Lungen-6 (KL-6) levels, and CT patterns categorized as usual interstitial pneumonia (UIP) or other patterns. Intraoperative variables were collected from anesthetic records and included the total oxygen volume administered, mean fraction of inspired oxygen (FiO_2_), operative time, and duration of anesthesia. The total intraoperative oxygen volume (L) was obtained directly from the automated clinical records recorded by the anesthesia delivery system. The system continuously measures and integrates the cumulative volumes of oxygen and air delivered to the patient. Therefore, the total oxygen volume was an actually measured value rather than a calculated estimate derived from fresh gas flow and FiO_2_ settings. This cumulative measurement automatically accounted for any variations in gas composition during both two-lung and one-lung ventilation.

The definition of AE in our study was based on the ATS/ERS/JRS/ALAT statement similar to the previous study: #1 worsening of dyspnea within one month, #2 evidence of hypoxemia, #3 new radiographic alveolar infiltrates, and #4 the absence of alternative explanations such as infection, pulmonary embolism, pneumothorax, or heart failure [14]. Two endpoints were evaluated: AE within 1-month (1mAE) and within 1-year (1yAE).

### Statistical analysis

Categorical variables were compared using Fisher’s exact test, and continuous variables were analyzed using the t-test or Welch’s t-test, as appropriate. Univariate and multivariate logistic regression analyses were performed to examine the predictive associations between pulmonary function and intraoperative anesthetic factors for 1mAE and 1yAE. The results are expressed as odds ratios (ORs) and 95% confidence intervals (CIs). The cutoff values for %VC, %FEV1, and %DLCO were set at 80% based on clinical guidelines [15, 16]. The optimal cutoff values of total intraoperative oxygen administration, mean FiO_2_, duration of anesthesia, and operative time for 1mAE and 1yAE were determined using the Youden index obtained from receiver operating characteristic (ROC) curve analysis to maximize discriminatory power. Multivariate logistic regression analysis was conducted using five different models. Each model incorporated different perioperative parameters while adjusting for baseline pulmonary function measurements. Variables were selected based on their clinical relevance and previous literature suggesting a potential impact on the postoperative outcomes. Simulation studies by Vittinghoff and McCulloch suggest that logistic regression models can yield valid estimates with five to nine events per predictor variable when effect sizes are strong, supporting the feasibility of our multivariable analysis. To address the potential for bias and instability in both univariate and multivariable models with a small number of events (specifically for 1mAE), we utilized Firth’s bias-reduced logistic regression [17, 18]. This method provides more reliable parameter estimates and mitigates the problem of separation in small cohorts. Furthermore, to evaluate the independence of total oxygen volume from surgical duration, an additional model including both parameters was analyzed using the Firth method.

Additionally, linear regression analysis was performed to examine the relationship between pulmonary function and intraoperative anesthetic factors. Statistical significance was set at *p* < 0.05. All analyses were conducted using JMP^®^ Student Edition software (version 18.2.0; SAS Institute Inc., Cary, NC, USA).

## Results

**Fig. 1:**
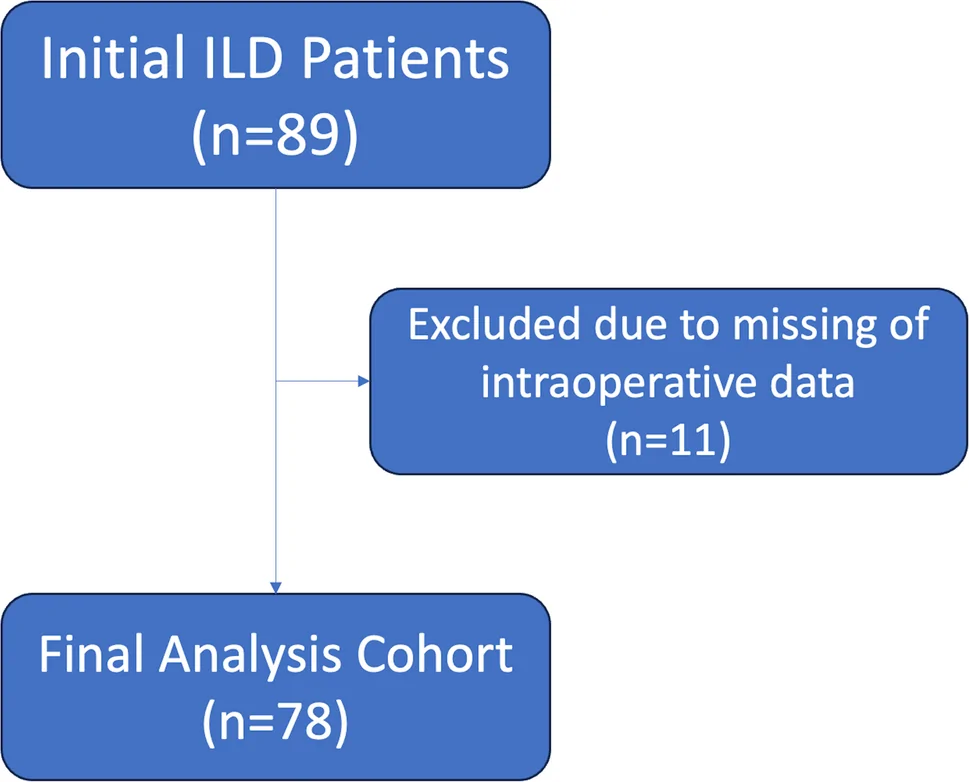
Flow diagram of patient recruitment. From 2014 to 2022, 89 patients with ILD underwent pulmonary surgery. Eleven are excluded due to missing intraoperative data, leaving 78 patients for analysis. ILD, Interstitial lung disease

Clinical data were initially obtained from 89 patients with ILD. However, 11 patients were excluded because of missing intraoperative data, resulting in a final analysis of 78 patients (Fig. 1).

### Patient characteristics

Eight patients (10.3%) had 1mAE and 14 (17.9%) had 1yAE after surgery. Among the 14 patients with 1yAE, 12 (85.7%) exhibited bilateral involvement, while only 2 (14.3%) had localized surgical-site AE. In the 1mAE cohort, 6 out of 8 patients (75.0%) presented with bilateral AE, and none experienced purely contralateral-site onset. No significant associations were observed between age, BMI, sex, smoking history, serum KL-6 levels, Radiological UIP pattern, perioperative Hb level, comorbidity, surgical procedure, and IPF (*p* > 0.05). The interstitial pneumonia observed in the group of patients without idiopathic pulmonary fibrosis happened to be connective tissue disease-associated ILD due to autoimmune diseases (rheumatoid interstitial, systemic scleroderma, dermatomyositis, and polymyositis). (Table 1).

**Table 1:** Baseline characteristics of patients with interstitial lung disease who underwent pulmonary surgery

	Total	1mAE (+)	1mAE (-)	*p*-value	1yAE (+)	1yAE (-)	*p*-value
	*n* = 78	*n* = 8	*n* = 70		*n* = 14	*n* = 64	
Age, year, mean (SD)	73.2 (11.6)	76.1 (6.7)	72.8 (12.1)	0.26	67.9 (21.1)	74.3 (8.2)	0.28
Male sex, n (%)	70 (89.7)	8 (100)	62 (88.6)	0.59	13 (92.9)	57 (89.1)	> 0.99
BMI, mean (SD)	22.4 (3.1)	23.1 (3.52)	22.3 (3.0)	0.54	22.6 (3.6)	22.3 (3.0)	0.77
Smoking				> 0.99			0.87
Current, n (%)	7 (9.0)	1 (12.5)	6 (8.57)		2 (14.3)	5 (7.8)	
Former, n (%)	53 (67.9)	6 (75)	47 (67.1)		9 (64.3)	44 (68.8)	
Never, n (%)	12 (15.4)	1 (12.5)	11 (15.7)		2 (14.3)	10 (15.6)	
Unknown, n (%)	6 (7.7)	0 (0)	6 (8.6)		1 (7.1)	5 (7.8)	
Comorbidity							
Asthma, n (%)	3 (3.9)	0 (0)	3 (4.3)	> 0.99	1 (7.1)	2 (3.1)	0.45
COPD, n (%)	43 (55.1)	4 (50)	39 (55.7)	> 0.99	7 (50)	36 (56.3)	0.77
Hb, g/dL, mean(SD)	13.2 (2.0)	12.5 (1.8)	13.3 (2.0)	0.22	12.7 (1.7)	13.4 (2.0)	0.24
Perioperative steroid use, n (%)	13 (16.7)	1 (12.5)	12 (17.1)	> 0.99	3 (21.4)	10 (15.6)	0.69
Surgical procedure, larger than segmentectomy, n (%)	36 (46.2)	5 (62.5)	31 (44.2)	0.46	6 (42.9)	30 (46.9)	> 0.99
Radiological UIP pattern (with honeycombing), n (%)	59 (75.6)	7 (87.5)	52 (74.3)	0.67	12 (85.7)	47 (73.4)	0.5
KL-6, U/mL, mean (SD)	736.7 (605.6)	684.8 (390.4)	744.2 (638.4)	0.72	897.6 (665.1)	690.7 (593.1)	0.31
IPF (%)	63 (80.8)	5 (62.5)	58 (82.9)	0.18	9 (64.3)	54 (84.4)	0.13

Abbreviations: *SD* standard deviation, *BMI* body mass index, *COPD* chronic obstructive pulmonary disease, *UIP* usual interstitial pneumonia, *KL-6* Krebs von den Lungen-6, *IPF* idiopathic pulmonary fibrosis, *1mAE* acute exacerbation occurring within 1 month, *1yAE* acute exacerbation occurring within 1 year

Regarding additional clinical parameters, home oxygen therapy was managed in only 1 patient (1.3%), and antifibrotic therapy was administered in 7 patients (9.0%; 6 received pirfenidone and 1 received nintedanib). Univariate analyses revealed no significant associations between these parameters and the development of acute exacerbations (all *p* > 0.05)

### Pulmonary function and anesthesia-related factors

The mean VC was 98.3% of the predicted value (standard deviation [SD], 18.3). The mean FEV1, DLCO, total intraoperative oxygen administration, FiO_2_, duration of anesthesia, and operative time were 87.7% (SD 15.8), 77.6% (SD 25.0), 673.7 L (SD 311.4), 81% (SD 13), 246.3 min (SD 98.4), and 164.9 min (SD 97.3), respectively.

Using ROC curves, we calculated the optimal cutoff values for 1mAE and 1yAE, which were identical: total intraoperative oxygen administration, 990 L; mean FiO_2_, 96%; duration of anesthesia, 303 min; and operative time, 206 min.

Univariate logistic regression (Table 2) showed that lower %VC (≤ 80%) was associated with both 1mAE and 1yAE. A lower %DLCO (≤ 80%) was associated with both 1yAE and 1mAE. Oxygen administration exceeding 990 L predicted both 1mAE and 1yAE. The mean FiO2 was associated with 1yAE. The extended duration of anesthesia and operative time were associated with 1mAE but not with 1yAE.

**Table 2:** Univariate logistic regression analysis for 1mAE and 1yAE using Firth’s bias-reduced method

	1mAE					1yAE				
		OR	95% CI		*p* value		OR	95% CI		*p* value
%VC	> 80%	1.00				> 80%	1.00			
	≤ 80%	2.46	1.16	5.26	0.024	≤ 80%	2.51	1.31	4.88	0.007
%FEV1	> 80%	1.00				> 80%	1.00			
	≤ 80%	1.82	0.88	3.79	0.12	≤ 80%	1.76	0.95	3.26	0.076
%DLCO	> 80%	1.00				> 80%	1.00			
	≤ 80%	2.34	1.05	7.38	0.027	≤ 80%	3.32	1.55	10.3	< 0.001
Total O_2_ volume	≤ 990 L	1.00				≤ 990 L	1.00			
	> 990 L	2.49	1.17	5.37	0.024	> 990 L	2.79	1.42	5.64	0.003
Mean FiO_2_	≤ 96%	1.00				≤ 96%	1.00			
	> 96%	1.86	0.84	3.93	0.15	> 96%	2.20	1.11	4.35	0.027
Duration of anesthesia	≤ 303 min	1.00				≤ 303 min				
	> 303 min	3.33	1.58	8.12	0.001	> 303 min	1.71	0.93	3.10	0.090
Operative time	≤ 206 min	1.00				≤ 206 min	1.00			
	> 206 min	3.05	1.46	7.41	0.003	> 206 min	1.56	0.86	2.81	0.15

Abbreviations: *VC* vital capacity, *FEV1* forced expiratory volume in 1 s, *DLCO* diffusing capacity of carbon monoxide, *FiO*_*2*_ fraction of inspired oxygen, *O*_*2*_ oxygen, *AE* acute exacerbation, *1mAE* acute exacerbation occurring within 1 month, *1yAE* acute exacerbation occurring within 1 year

All odds ratios and 95% confidence intervals were estimated using Firth’s bias-reduced logistic regression to ensure the stability of the model in the context of a small number of outcome events

### Multivariate analysis

In the multivariate analysis (Table 3), in Model 1, total oxygen administration exceeding 990 L showed a borderline association with 1mAE and was a significant predictor of 1yAE. In Model 2, the mean FiO_2_ was not a significant predictor of either 1mAE or 1yAE. In Models 3 and 4, the extended duration of anesthesia and operative time were significantly associated with 1mAE but not with 1yAE. In Model 5, total oxygen administration exceeding 990 L was significantly associated with 1yAE but not with 1mAE.

**Table 3:** Multivariable logistic regression analysis for 1mAE and 1yAE using Firth’s bias-reduced method

		1mAE					1yAE				
			OR	95% CI		*p* value		OR	95% CI		*p* value
Model 1	Total O_2_ volume	≤ 990 L	1.00				≤ 990 L	1.00			
		> 990 L	2.12	0.93	4.99	0.071	> 990 L	2.60	1.21	6.12	0.012
	VC	> 80%	1.00				> 80%	1.00			
		≤ 80%	2.50	1.10	5.91	0.028	≤ 80%	2.40	1.07	5.70	0.030
	%DLCO	> 80%	1.00				> 80%	1.00			
		≤ 80%	2.00	0.81	6.57	0.12	≤ 80%	2.90	1.22	10.2	0.008
Model 2	Mean FiO_2_	≤ 96%	1.00				≤ 96%	1.00			
		> 96%	1.56	0.65	3.71	0.35	> 96%	1.90	0.87	4.25	0.10
	%VC	> 80%	1.00				> 80%	1.00			
		≤ 80%	2.61	1.15	6.11	0.021	≤ 80%	2.53	1.13	6.09	0.021
	%DLCO	> 80%	1.00				> 80%	1.00			
		≤ 80%	1.74	0.73	5.6	0.19	≤ 80%	2.38	1.06	7.48	0.023
Model 3	Duration of anesthesia	≤ 303 min	1.00				≤ 303 min	1.00			
		> 303 min	3.33	1.46	9.32	0.003	> 303 min	1.76	0.85	3.76	0.13
	%VC	> 80%	1.00				> 80%	1.00			
		≤ 80%	2.48	1.01	6.52	0.044	≤ 80%	2.58	1.21	5.89	0.012
	%DLCO	> 80%	1.00				> 80%	1.00			
		≤ 80%	2.37	0.94	8.14	0.051	≤ 80%	3.09	1.37	9.92	0.003
Model 4	Operative time	≤ 206 min	1.00				≤ 206 min	1.00			
		> 206 min	3.11	1.37	8.68	0.011	> 206 min	1.61	0.79	3.37	0.18
	%VC	> 80%	1.00				> 80%	1.00			
		≤ 80%	2.57	1.06	6.73	0.034	≤ 80%	2.62	1.23	5.95	0.011
	%DLCO	> 80%	1.00				> 80%	1.00			
		≤ 80%	2.31	0.93	7.83	0.056	≤ 80%	3.03	1.36	9.64	0.003
Model 5	Total O_2_ volume	≤ 990 L	1.00				≤ 990 L	1.00			
		> 990 L	1.53	0.63	3.59	0.36	> 990 L	2.29	1.09	4.93	0.029
	Duration of anesthesia	≤ 303 min	1.00				≤ 303 min	1.00			
		> 303 min	1.63	0.41	20.0	0.38	> 303 min	1.29	0.32	15.9	0.63
	Operative time	≤ 206 min	1.00				≤ 206 min	1.00			
		> 206 min	1.68	0.14	6.79	> 0.99	> 206 min	1.61	0.09	3.82	> 0.99

Abbreviations: *OR* odds ratio, *CI* confidence interval, *VC* vital capacity, *DLCO* diffusing capacity of carbon monoxide, *O*_*2*_ oxygen, *AE* acute exacerbation, *1mAE* acute exacerbation occurring within 1 month, *1yAE* acute exacerbation occurring within 1 year

All odds ratios and 95% confidence intervals were estimated using Firth’s bias-reduced logistic regression to ensure the stability of the model in the context of a small number of outcome events

A %VC ≤ 80% consistently remained a significant predictor of both 1mAE and 1yAE across all models. In 1–4 models, a %DLCO ≤ 80% was a significant risk factor for 1yAE but not for 1mAE.

**Fig. 2:**
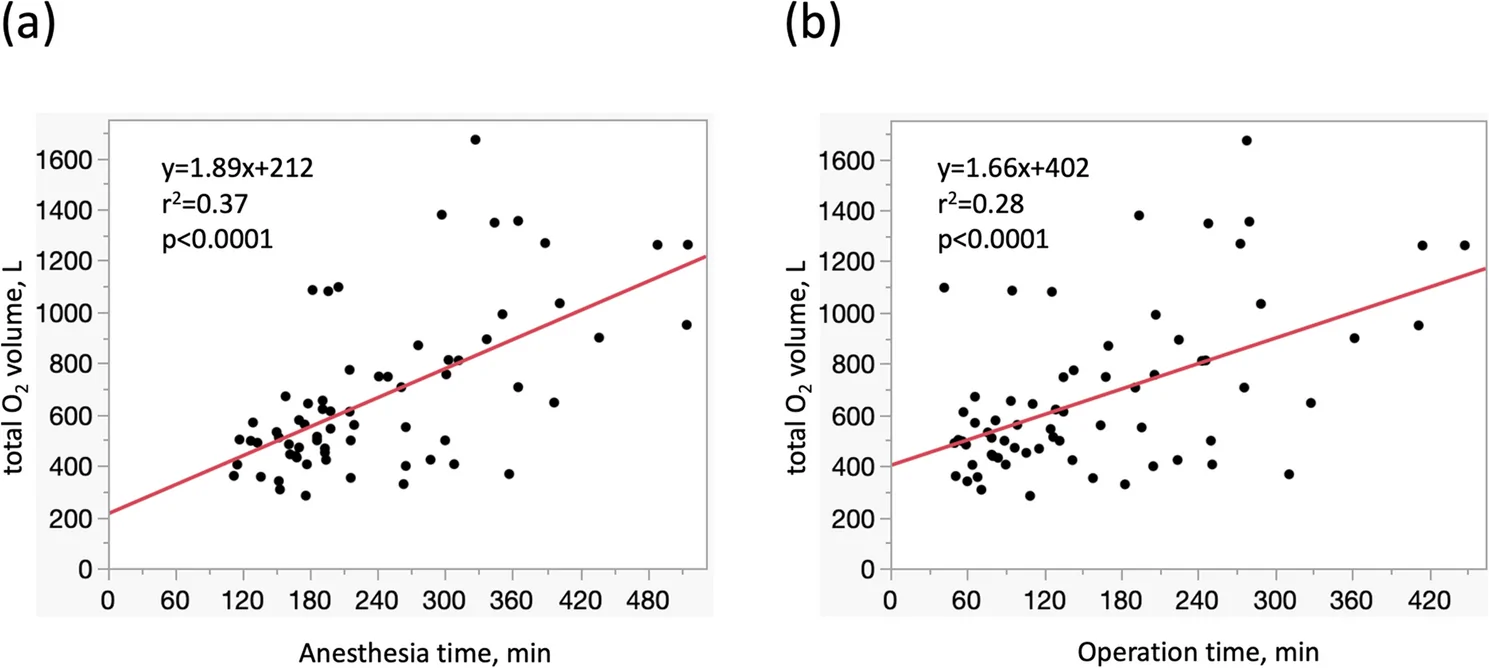
Linear regression analyses showing the association between total intraoperative oxygen volume and surgical time parameters. **a** A significant positive correlation is observed between the total oxygen volume and duration of anesthesia (*y* = 1.89*x* + 212, *r*^*2­­­*^ = 0.37, *p* < 0.0001). **b** A similar correlation is found between total oxygen volume and operative time (*y* = 1.66*x* + 402, *r*^*2­­­*^ = 0.28, *p* < 0.0001)

In addition, linear regression analyses revealed significant positive correlations between the total intraoperative oxygen volume and duration of anesthesia (Fig. 2a; *y* = 1.89*x* + 212, *r*^*2­­­*^ = 0.37, *p* < 0.0001) and operative time (Fig. 2b; *y* = 1.66*x* + 402, *r*^*2­­­*^ = 0.28, *p* < 0.0001). Furthermore, linear regression analyses showed no significant correlations between preoperative pulmonary function parameters (%VC, %FEV1, and %DLCO) and intraoperative factors, including anesthesia duration, operative time, and total oxygen volume (all *p* > 0.05).

## Discussion

As described in the Results section, %VC was significantly associated with both 1mAE and 1yAE. %DLCO, and total intraoperative oxygen volume > 990 L were identified as significant predictors of 1yAE. These variables may serve as useful markers for identifying patients at an elevated risk. Previous studies have also suggested that pulmonary function is a predictor of AE [3, 19], and excessive oxygen exposure can lead to alveolar injury [20]. In clinical practice, based on experience, it is often advised to minimize oxygen administration in patients with ILD whenever possible. Our findings are consistent with this hypothesis.

Linear regression analysis showed that operative time and duration of anesthesia were naturally correlated with total intraoperative oxygen administration volume. However, our subsequent multivariable models across the 1-month and 1-year endpoints revealed a distinctly nuanced, time-dependent risk pattern. In the short-term dimension, prolonged operative time and extended duration of anesthesia—which should primarily reflect the overall magnitude of acute surgical trauma and physical stress—displayed pronounced associations with 1mAE. This acute surgical insult upregulates pro-inflammatory cytokines, such as interleukin (IL)-6 and tumor necrosis factor (TNF)-α [21], and promotes the localized generation of reactive oxygen species (ROS) [22]. These molecular events can induce acute alveolar epithelial injury and severe immune dysregulation, thereby predisposing susceptible patients with ILD to early-onset acute postoperative exacerbations.

In sharp contrast, when evaluating the long-term prognosis (1yAE) through the direct intraoperative interaction model (Model 5), these time-dependent operational parameters lost their prognostic power, whereas total intraoperative oxygen administration > 990 L emerged as a robust, independent predictor (OR 2.29, 95% CI: 1.09–4.93, *p* = 0.029). While the total oxygen volume exhibited a marginal association with 1mAE in Model 1 (OR 2.12, 95% CI: 0.93–4.99, *p* = 0.071), the consistent trend observed across both early and late postoperative phases reinforces the clinical importance of adopting a conservative, “oxygen-protective” strategy to minimize cumulative oxidative burden.

Taken together, these findings suggest that cumulative intraoperative oxygen exposure may be associated with a distinct pathological process in fibrotic lungs that extends beyond the acute phase. While immediate physical surgical stress potentially dictates the incidence of short-term AEs, the concurrent cumulative oxygen load can initiate a progressive, subacute-to-chronic oxidative cascade that independently drives long-term pulmonary deterioration. While this retrospective study cannot establish a direct causal link, hyperoxia could potentially contribute to alveolar epithelial injury through excessive production of ROS, which activates redox-sensitive signaling pathways, such as nuclear factor kappaB and mitogen-activated protein kinase [23, 24], and promotes the release of pro-inflammatory cytokines (e.g., IL-1beta, IL-6, and TNF-alpha) [25, 26].

This cascade should be particularly critical during one-lung ventilation (OLV), where the ventilated lung faces a “double-hit” of physical overdistension (volutrauma) and high-concentration oxygen stress. In our cohort, the majority of AEs (75.0% for 1mAE and 85.7% for 1yAE) were bilateral and diffuse. This clinical presentation suggests that postoperative AE-ILD is a generalized pulmonary process driven by cumulative oxidative stress rather than a localized insult during a specific ventilation phase. Therefore, monitoring the total whole-procedure oxygen exposure should serve as a highly practical surrogate for the overall oxidative burden. This mechanical-oxidative synergy may well amplify hyperoxia-induced ROS generation, which hyperactivates TGF-beta-mediated fibroproliferative pathways and epithelial-mesenchymal transition [27, 28], ultimately precipitating the diffuse alveolar damage characteristic of AE. Consequently, cumulative oxygen toxicity can exert both immediate inflammatory and delayed fibrotic effects through these distinct but overlapping molecular mechanisms.

In the present study, variables such as serum KL-6 levels, preoperative steroid use, and surgical procedure type did not demonstrate significant associations with AE development. These findings, while likely constrained by our sample size, suggest that cumulative intraoperative oxygen exposure and baseline pulmonary function (%VC and %DLCO) are more robust predictors of AE than these conventional factors in our specific cohort. For instance, preoperative steroid use likely reflects the clinical management of specific ILD subtypes rather than serving as a direct independent risk factor for AE.

One of the strengths of our study is that, to the best of our knowledge, this is the first study to demonstrate that total intraoperative oxygen administration may contribute to postoperative AE in patients with ILD using real-world clinical data. Furthermore, we extended this finding by showing that total oxygen volume, rather than FiO_2_ alone, may be a more meaningful predictor. Therefore, oxygen toxicity may have a cumulative effect. Our results support the hypothesis that cumulative oxygen exposure leads to alveolar damage via oxidative stress. Based on these findings, the development of optimal intraoperative oxygen protocols for patients with ILD may be beneficial and could potentially improve the safety of lung surgery in this high-risk population.

Our findings also highlight a potential threshold effect of oxygen toxicity in fibrotic lungs. While the mean FiO_2_ represents the intensity of exposure, the total oxygen volume potentially reflects the cumulative oxidative burden. Patients with ILD often have reduced antioxidant reserves [29], which can make the alveolar epithelium particularly susceptible to ROS-mediated injury triggered by hyperoxia [30]. This “second hit” from cumulative oxygen load, superimposed on the “first hit” of surgical stress, may precipitate the diffuse alveolar damage characteristic of AE [31]. Therefore, the identified cutoff value (approximately 990 L) may offer a practical benchmark for risk stratification. However, it should be noted that this threshold was derived from our specific, relatively small cohort. This numerical value should be interpreted as an indicative signal highlighting the potential risk of cumulative oxygen toxicity rather than a definitive clinical rule. Our findings emphasize the clinical importance of the “as low as reasonably achievable” principle for intraoperative oxygen administration, rather than adhering to a specific numerical limit. Clinically, this suggests that anesthetic management for ILD patients should focus not only on avoiding high FiO_2_, but also on limiting total gas flow and the duration of exposure, similar in principle to lung-protective ventilation, but applied to oxygen dosing. For preoperative planning, preparing alternative intraoperative strategies and monitoring oxygen volume in real time may help guide adjustments when procedures become more invasive than expected, thereby reducing the risk.

To implement these findings into clinical practice, a multifaceted approach to “oxygen-protective” anesthesia is required, particularly for high-risk patients with low preoperative pulmonary function. First, optimizing recruitment maneuvers and applying appropriate levels of positive end-expiratory pressure—within a range that does not compromise systemic hemodynamics—can improve ventilation-perfusion matching. This allows for a reduction in the required FiO_2_ and fresh gas flow while maintaining adequate systemic oxygenation. Second, efforts to minimize operative and anesthesia durations through meticulous preoperative planning and efficient surgical techniques directly contribute to reducing the cumulative oxygen burden. By integrating these strategies, clinicians can minimize oxygen toxicity without compromising patient safety, shifting the intraoperative goal from “liberal oxygenation” to “conservative and optimized oxygen dosing” in the management of ILD.

### Limitations

This study had several limitations. First, its retrospective design may have introduced selection bias and limited causal inferences. Second, the sample size was relatively small, which may have affected the statistical power of the analysis. Third, intraoperative oxygen administration was assessed as a cumulative volume rather than as a continuous variable, which may not fully capture the dynamic effects of oxygen exposure. Fourth, due to the retrospective nature of this study and the limitations of our anesthesia records, we were unable to separate the exact oxygen volumes delivered during the distinct phases of OLV and two-lung ventilation. However, given that the overwhelming majority of AE cases in our cohort presented with bilateral lung injury, evaluating the total perioperative cumulative oxygen volume remains a physiologically sound and holistic marker of the overall oxidative burden. Finally, this study was conducted at a single institution. External validation in a larger multicenter cohort is required to generalize our findings.

## Conclusions

Our findings suggest that total oxygen administration during surgery (rather than FiO_2_), combined with %VC and %DLCO values, serves as an independent predictor of long-term (1-year) postoperative AE in patients with ILD. Although its independent association with short-term (1-month) AE remained marginal, the overall trend highlights the clinical importance of monitoring and optimizing cumulative oxygen exposure throughout the perioperative period. Further studies on intraoperative oxygen management may contribute to improved surgical planning and perioperative safety in patients with ILD.

## Data Availability

The datasets used and/or analyzed during the current study are available from the corresponding author on reasonable request.

## Abbreviations

1mAE: Acute exacerbations occurring within 1 month
1yAE: Acute exacerbations occurring within 1 year
AE: Acute exacerbation
BMI: Body mass index
CI: Confidence interval
COPD: Chronic obstructive pulmonary disease
CT: Computed tomography
%DLCO: %diffusing capacity for carbon monoxide
%FEV1: %forced expiratory volume in 1 s
FiO_2_: Fraction of inspired oxygen
IL: Interleukin
IPF: Idiopathic pulmonary fibrosis
ILD: Interstitial lung disease
KL-6: Krebs von den Lungen-6
OR: Odds ratio
ROS: Reactive oxygen species
SD: Standard deviation
TNF: Tumor necrosis factor
UIP: Usual interstitial pneumonia
%VC: %vital capacity
